# An updated view into the cell cycle kinetics of human T lymphocytes and the impact of irradiation

**DOI:** 10.1038/s41598-022-11364-9

**Published:** 2022-05-10

**Authors:** Evi Duthoo, Anne Vral, Ans Baeyens

**Affiliations:** 1 Radiobiology Group, Department of Human Structure and Repair, Ghent University, Ghent, Belgium; 2 Cancer Research Institute Ghent (CRIG), Ghent, Belgium

**Keywords:** Checkpoints, Flow cytometry

## Abstract

Even though a detailed understanding of the proliferative characteristics of T lymphocytes is imperative in many research fields, prior studies have never reached a consensus on these characteristics, and on the corresponding cell cycle kinetics specifically. In this study, the general proliferative response of human T lymphocytes to phytohaemagglutinin (PHA) stimulation was characterized using a carboxyfluorescein succinimidyl ester-based flow cytometric assay. We were able to determine when PHA-stimulated T lymphocytes complete their first division, the proportion of cells that initiate proliferation, the subsequent division rate of the cells, and the impact of irradiation on these proliferative properties. Next, we accurately visualized the cell cycle progression of dividing T lymphocytes cultured in whole blood using an adapted 5-ethynyl-2’-deoxyuridine pulse-chase method. Furthermore, through multiple downstream analysis methods, we were able to make an estimation of the corresponding cell cycle kinetics. We also visualized the impact of X-rays on the progression of the cells through the cell cycle. Our results showed dose-dependent G2 arrest after exposure to irradiation, and a corresponding delay in G1 phase-entry of the cells. In conclusion, utilizing various flow cytometric assays, we provided valuable information on T lymphocyte proliferation characteristics starting from first division to fully dividing cells.

## Introduction

A thorough understanding of the proliferative characteristics of cells forms a cornerstone of basic, translational, and clinical biological research. Human lymphocytes are widely used in various research fields and detailed knowledge about their proliferation properties and cell cycle kinetics is often key. Many studies have been performed into lymphocyte proliferation, especially between the 1960 and 1990s^1–6^. However, to present day, the available information remains quite conflicting. Various studies described different total cell cycle durations, ranging from 10 to 24 h. Significant variations were particularly reported for the G1 cell cycle phase^1–4,6^. These discrepancies may be explained by differences in culture methods, mitogen stimuli (often phytohaemagglutinin (PHA)), or even the employed proliferation assays. The various methods used in these studies, such as tritiated thymidine-based assays, are now seldomly utilized due to the accessibility of better alternatives.

Human lymphocytes are often used in diverse research fields, partly due to their considerable potential as an easily accessible source of cells that can be collected in a minimally invasive manner. In the context of cancer chemotherapy, the development of drugs that target cell cycle-specific proteins has proven to be of highly clinical importance^7^. Also in the immunology field, insight into the proliferative characteristics of T lymphocytes can aid in vitro studies where the functionality or the distinct cytokine profile of T cells is evaluated^8,9^. Furthermore, understanding the impact of genotoxic agents, such as irradiation, on the cell cycle and division abilities of these cells plays a crucial role in many radiobiology studies^10,11^. However, our outdated knowledge on lymphocyte proliferation and cell cycle kinetics, and correspondingly the underlying regulatory mechanisms, may be impediment for these research fields.

Numerous techniques are currently available to study both cell proliferation and cell cycle kinetics. These methods are based on various aspects of the cell cycle and can be divided into: (1) cytoplasmic proliferation dye-based assays; (2) nucleoside-analogue incorporation assays; (3) cell cycle-associated protein assays; and (4) indirect methods based on cell counting, viability, and metabolic activity^12^. The use of cytoplasmic proliferation dyes, classically the green fluorescence dye carboxyfluorescein succinimidyl ester (CFSE), is an effective method to monitor cell proliferation. CFSE is cell permeable and has the ability to covalently bind to cytoplasmatic components, resulting in a uniform bright fluorescence signal. Upon cell division, the dye is equally distributed between daughter cells resulting in a decrease of fluorescence intensity. Cell proliferation can subsequently be traced for up to eight divisions using flow cytometry^13,14^. CFSE is a powerful tool to quantitatively analyze cell divisions, both in vivo and in vitro, and is routinely used in both research and clinical settings^15–18^.

To study the cell cycle, nucleoside-analogue incorporation assays are often utilized. During replication, nucleoside-analogues can be incorporated into cellular DNA, tagging the cells in S phase, followed by immunofluorescence or flow cytometric analysis. So far, the most commonly used thymidine-analogues are tritiated thymidine ([^3^H]TdR) and 5-bromo-2’-deoxyuridine (BrdU)^1,3^. However, both techniques, despite their sensitivity and reliability, are associated with several imperfections. The tritiated thymidine method uses a radioactively labeled thymidine, which requires careful handling and expensive detection devices. Furthermore, this technique quantifies overall cell division, but does not allow for single cell monitoring^12^. BrdU, a non-radioactive alternative, relies on an antibody-based detection method that requires DNA denaturation by heat or acid treatment^19^. However, this can results in modifications of cellular epitopes, degradation of the DNA structure and can cause variability in the staining intensity^20^. Recently, a new thymidine-analogue 5-ethynyl-2’-deoxyuridine (EdU) has been developed as an alternative to the tritiated thymidine and BrdU method^21^. This assay is based on copper-catalyzed Click-iT technology for the detection of EdU incorporation and does not require DNA denaturation. The EdU method harbors several major advantages, such as rapidity and simplicity, compared to alternative thymidine-analogues. Combining thymidine-analogue incorporation methods with traditional cell cycle analysis, based on the measurement of DNA content using propidium iodide or DAPI, allows for highly efficient flow cytometry-based proliferation assays^22^.

The objectives of the present study were to examine the proliferative characteristics, cell cycle progression, and kinetics of the cell cycle phases of cultured human T lymphocytes. These properties were examined using the CFSE proliferation assay and the EdU pulse-chase method, for both non-irradiated and irradiated cells. T lymphocytes were stimulated with the mitogen PHA and, utilizing CFSE, we subsequently determined when the cells complete their first division, the number of cells that initiate proliferation, and the subsequent division rate of these cells. Using an adapted EdU pulse-chase method, the progression of PHA-stimulated T lymphocytes through the different phases of the cell cycle was visualized. Subsequently, using different downstream analysis methods, we were able to estimate the corresponding cell cycle phase kinetics. This adapted method allowed us to chase T lymphocytes cultured in whole blood, which provided us with a model that better represents the in vivo situation.

Our findings reveal various proliferative properties that are characteristic for T lymphocytes stimulated with PHA. Moreover, we were able to make an estimation of the cell cycle kinetics of T lymphocytes cultured in whole blood and uncover the unique effects of X-rays on the cell cycle progression of these cells.

## Materials and methods

### Sample collection

Heparinized peripheral blood samples were obtained from healthy volunteers (n = 14) with an age between 24 and 55 years. Informed consents were obtained from the blood donors. This study was approved by the Ethics Committee of Ghent University Hospital (reference no. 2019/1565). Blood donors in this study had no known previous exposure to medication, radiotherapy, or substances that could affect the results of this study. All experiments were performed according to the relevant guidelines and regulations.

### Irradiation procedure

Irradiations were performed using the Small Animal Radiation Research Platform (SARRP) (Xstrahl, Camberley, UK) at Infinity Lab, Ghent University. Cultures were irradiated using X-rays (220 kV, 13 mA, 0.15 mm copper filter) at a dose rate of 3 Gy/min.

### CFSE proliferation assay for isolated PBMCs

Peripheral blood mononuclear cells (PBMCs) were isolated from heparinized blood samples using Lymphoprep (Axis-Shield, Dundee, UK) gradient centrifugation. Following isolation, PBMCs were resuspended in PBS at a density of 1 × 10^6^ cells per ml. Cell proliferation was examined using CFSE (CellTrace CFSE Cell Proliferation Kit, Thermo Fisher Scientific, Waltham, MA, USA) based on Quah et al.^13^ Cells were labeled with CFSE at a final concentration of 1 µM and immediately vortexed to ensure rapid and homogeneous labeling. The cells were incubated for 10 min at 37 °C. Unbound CFSE was quenched by the addition of cold PBS with 1% fetal calf serum (FCS). The cells were washed twice with PBS containing 1% FCS and then seeded in a cell suspension 48 well plate (Greiner Cellstar, Sigma-Aldrich, Saint Louis, MO, USA) at a concentration of 250,000 cells in 500 µl growth medium consisting of RPMI-1640 medium supplemented with 10% FCS, 1% sodium pyruvate, 0.1% β-mercapthoethanol, penicillin (50 U/ml), and streptomycin (50 mg/ml), all from Gibco (Thermo Fisher Scientific, Waltham, MA, USA). Immediately following CFSE labeling and seeding, the PBMCs were irradiated with 1 and 2 Gy of X-rays. Sham-irradiated samples (0 Gy) were included. Subsequently, the cells were stimulated with phytohaemagglutinin-M (PHA-M, 5 µl/ml) (Sigma-Aldrich, Saint Louis, MO, USA). After 0–96 h of culture, with 24-h intervals, the cells were harvested, washed twice with PBS, and analyzed by flow cytometry. Cell viability was assessed using Propidium Iodide (PI). Additional flow cytometric analyses, immediately following CFSE labeling, indicates a labeling efficiency that exceeds 99%.

### EdU pulse-chasing method for whole blood cultures

**Figure 1:**
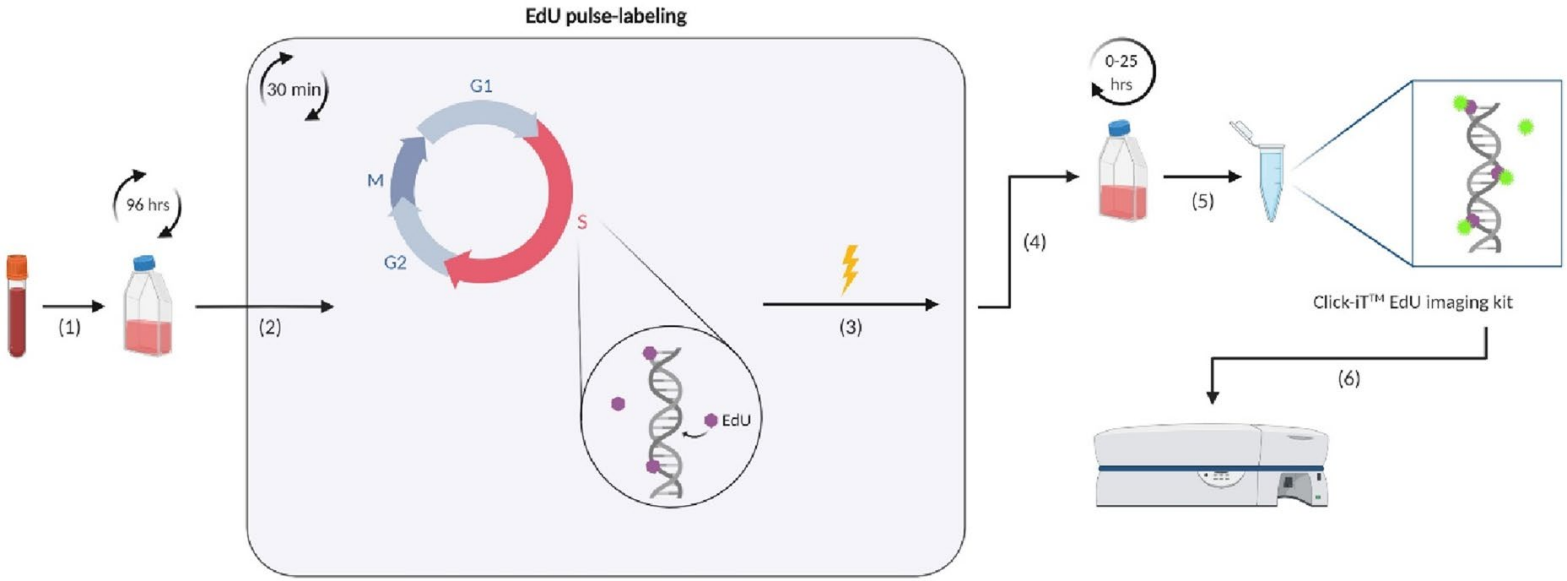
Schematic overview of the EdU pulse-chasing method for tracking T lymphocytes cultured in whole blood. (**1**) Whole blood is cultured and lymphocyte cell division is stimulated using PHA. (**2**) After 96 h of culture, the proliferating cells are pulse-labeled with 10 µM EdU for 30 min; and (**3**) during pulse-labeling, the cells are irradiated in vitro with 2 or 4 Gy of X-rays. (**4**) EdU pulse-chasing is performed by culturing the cells from 0 up to 25 h and chasing them over time. (**5**) Following red blood cell lysis, the lymphocytes are fixed and then stained using the Click-iT EdU imaging kit. (**6**) The samples are measured using flow cytometry and analyzed using FlowJo software.

Whole blood cultures were set-up by adding 0.5 ml of fresh heparinized blood to 4.5 ml of RPMI-1640 medium supplemented with 10% FCS, penicillin (50 U/ml), and streptomycin (50 mg/ml). Cell division was stimulated using PHA-M (20 µl/ml). EdU labeling and staining (Click-iT EdU Alexa Fluor 555 Imaging Kit, Thermo Fisher Scientific, Waltham, MA, USA) was performed according to Sun et al.^23^ After 96 h of culture, dividing T lymphocytes were pulse-labeled with 10 µM EdU (dissolved in DMSO as per manufacturer’s instructions) for 30 min at 37 °C and, during pulse-labeling, the cells were irradiated with 2 and 4 Gy of X-rays. Sham-irradiated samples (0 Gy) were included. For EdU pulse-chasing (Fig. 1), the cells were washed twice with prewarmed PBS, supplemented with EdU-free prewarmed medium, and cultured at 37 °C, 5% CO_2_. The cells were chased over time for hours ranging from 0 to 25 h.

After varying culture times, the erythrocytes were lysed using lysis buffer. This was done in order to simplify the flow cytometric evaluation of the T lymphocytes. Subsequently, the cells were washed twice with PBS and the pellet was fixed in 3% paraformaldehyde (VWR International, Wayne, PA, USA) for 20 min, at room temperature (RT). The cells were washed twice with PBS containing 1% FCS and permeabilized for 10 min with ice-cold 0.2% Triton X-100 in PBS (Sigma-Aldrich, Saint Louis, MO, USA). Next, the cells were incubated at RT in the dark for 30 min with 100 µl of the Click-iT reaction cocktail, prepared as per manufacturer’s instructions. After washing twice with PBS, DNA was stained using 100 µl of DAPI solution (1 µg/ml) (Sigma-Aldrich, Saint Louis, MO, USA) and the cells were analyzed by flow cytometry. Additional non-irradiated and irradiated whole blood cultures — supplemented with DMSO without EdU — were included to determine the possible toxic effect of EdU on the cell cycle kinetics.

### Analysis by flow cytometry

Flow cytometry was performed using a BD LSR II (BD Biosciences, San Jose, CA, USA) equipped with four lasers (488 nm, 633 nm, 405 nm, and 355 nm). Cell debris and doublets were excluded from the analysis using FSC-A versus SSC-A and FSC-A versus FSC-H when appropriate. An average of 10,000 events was collected per sample after these gatings, in a slow rate mode to avoid doublets^24^. Additional gatings were applied on both the CFSE proliferation and EdU pulse-chase data according to the analysis (Supplementary Figs. S1 and S2). Minor differences between donor samples were observed. This was included in subsequent sample analyzes by adjusting the gatings accordingly. It is important to note that the whole lymphocyte fraction was analyzed according to the gating strategy shown in Supplementary Figs. S1 and S2 without an extra T lymphocyte staining. A minor fraction of B lymphocytes and natural killer (NK) cells might thus still be present in the sample. However, as PHA stimulates proliferation of specifically T lymphocytes, all analyzed proliferating cells are expected to be T lymphocytes^25–27^.

### Quantitative data analysis

Further quantitative data analyses were performed using both the FlowJo Software v10.7 (BD Life Sciences) (https://flowjo.com) and GraphPad Prism v9.2.0 for Windows (GraphPad Software, San Diego, CA, USA) (https://graphpad.com). Statistical analysis was performed using GraphPad Prism. The precursor frequency (PF) and lymphocyte proliferation index (LPI) of each CFSE sample was determined using the Proliferation Tool in the FlowJo Software package. This tool calculates the PF and LPI as described by Wells et al. and Roederer^15,28^. Significance of differences between both the PFs and the PIs of the different irradiation doses was determined for each time point using a Kruskal–Wallis test followed by a Dunn's post hoc test.

The Cell Cycle Analysis tool in the FlowJo Software package was utilized to compare T lymphocyte cell cycle phase distributions of both EdU-labeled and non-labeled whole blood cultures in order to determine EdU-toxicity (Supplementary Fig. S3). The EdU pulse-chase data was quantified as the percentage of EdU-positive cells in the G2/M phase, G1 phase, or in G2 arrest, respectively. The duration of the S phase was defined as the full-width-at-half-maximum (FWHM) of the hourly percentage of EdU-positive cells in the G2/M phase^29,30^. The FWHM was calculated using the SymPy mathematics Python package in a custom Python script^31^, starting from the equation parameters of a cubic fit applied in GraphPad Prism. This fit was applied for each sample individually, resulting in a FWHM value for each sample. Using the relative movement (RM) technique as described by Begg et al., the estimated duration of the S phase was verified (Supplementary Fig. S4)^32^. The G2/M phase duration was defined as the time interval between the start of influx of cells into the G2/M phase and the time needed to reach a maximum number of cells in G2/M phase^33^. These time points were determined from the cubic fit equation of each sample individually. From the lymphocyte proliferation indexes of all time points, the total cell cycle time (Tc) was estimated (Supplementary Table S1).

## Results

### Examining the proliferative characteristics of isolated T lymphocytes using the CFSE assay

#### T lymphocytes complete a first division between 48 and 72 h of culture with PHA

**Figure 2:**
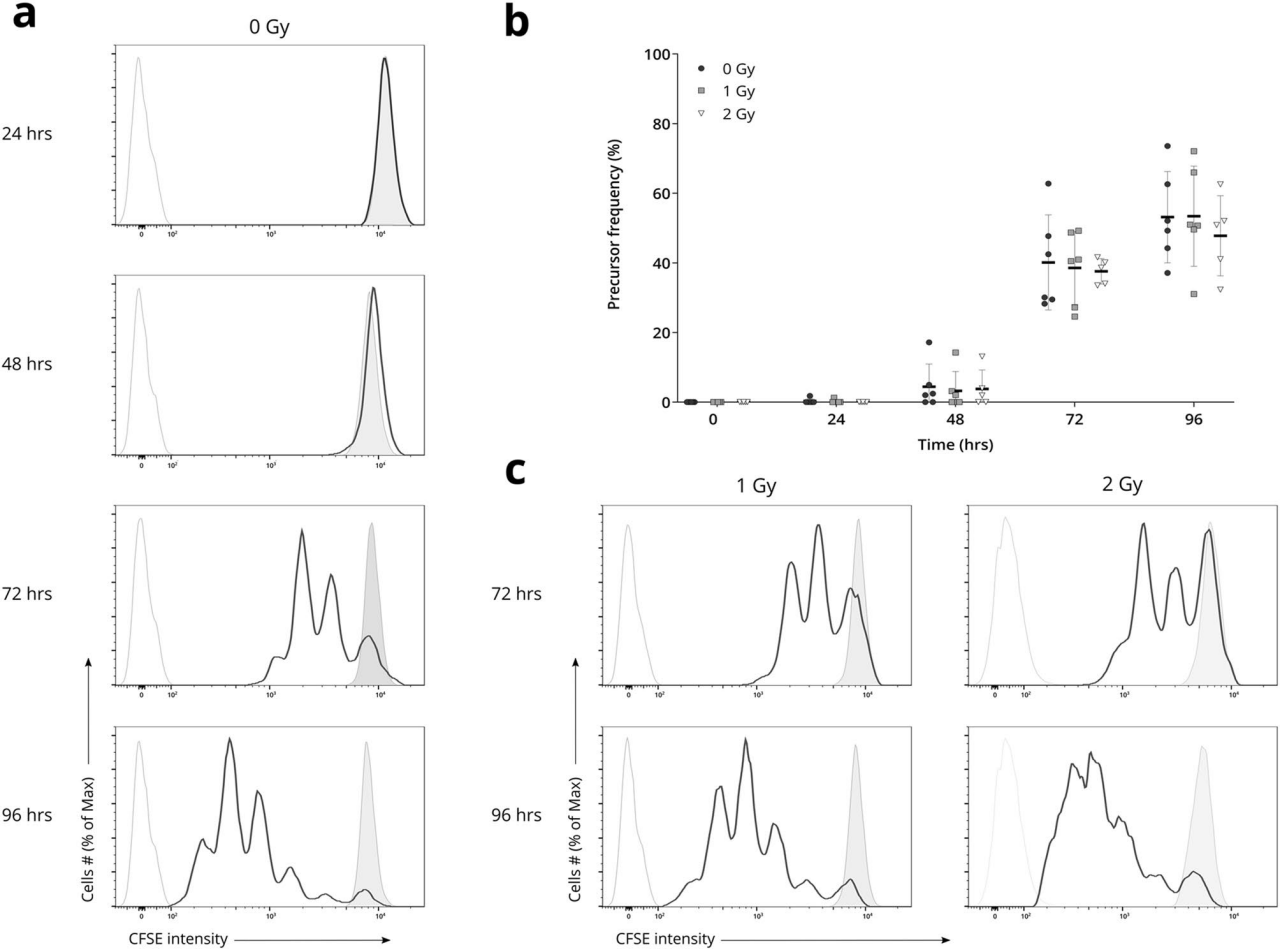
Proliferation analysis of CFSE-labeled T lymphocytes after PHA stimulus. T lymphocytes were exposed to 0, 1, and 2 Gy of 220 kV X-rays and cell proliferation was examined at 0, 24, 48, 72, and 96 h. (**a**) Representative example of CFSE fluorescence profiles of live non-irradiated T lymphocytes. The open black histograms show stimulated CFSE-labeled cells. The solid grey histograms show unstimulated CFSE-labeled control samples. The open grey histograms show the autofluorescence of stimulated control samples, not labeled with CFSE. (**b**) The precursor frequency (PF) of CFSE-labeled cells for each irradiation dose, at various time points. Individual datapoints of 6 independent experiments are shown, with the mean ± standard deviation. No statistically significant differences were found (*p* > 0.05) between irradiation doses. (**c**) Representative example of CFSE fluorescence profiles of live irradiated T lymphocytes visualized as described for (a).

To examine when T lymphocytes complete their first division after PHA stimulus, isolated PBMCs were homogeneously labeled with CFSE, which initially results in a bright and uniform peak of CFSE-positive cells (Fig. 2a). Proliferation of the CFSE-labeled cells results in the sequential halving of the fluorescence signal with each division, as illustrated in Supplementary Fig. S5. Note that the traceable number of divisions is limited by the autofluorescence level of the unstained activated control sample^13^. Upon stimulation with PHA, T lymphocyte proliferation was examined at 0, 24, 48, 72, and 96 h. Furthermore, the effect of irradiation on the duration of the first division was examined by exposing the cells to 1 and 2 Gy of X-rays before stimulation.

Our results show that a first T lymphocyte division is completed between 48 and 72 h after PHA stimulation (Fig. 2a). When examining the amount of performed divisions, we can see that after 72 h, some cells have divided up to three times, while other cells remain undivided throughout this same period of time (Fig. 2a). This indicates moderate asynchrony in the cell proliferation kinetics of T lymphocytes. Our findings also show that irradiation of the T lymphocytes before stimulation has no visible impact on the average time needed for a first division (Fig. 2c). Noteworthy, the undivided T lymphocytes continue to fluoresce brightly after 96 h, confirming dye stability over the cultured period of time.

#### The proportion of T lymphocytes completing a first division is not altered by exposure to irradiation

To compare the proportion of dividing cells between non-irradiated and irradiated T lymphocytes, we determined the precursor frequency (PF) for all time points and each irradiation dose. The PF is defined as the fraction of cells of the starting population that divided at least once during the culture period. This provides us with a good measurement to determine the initial proliferation response of the cells, without being biased by any potential differences in the division rate.

For each irradiation dose, a major increase in average PF could be observed between 48 and 72 h, confirming our previous observations regarding the average time needed for the cells to start proliferating (Fig. 2b and Supplementary Table S2). Notably, no significant effect of irradiation on the PF could be observed (*p* > 0.05), indicating that exposure to X-rays prior to PHA stimulation has no influence on the number of cells that initiate proliferation. Interestingly, after 96 h of stimulation, an average of 46.86%, 46.60%, and 52.52% of the cells have not started proliferating after exposure to 0, 1, and 2 Gy of X-rays respectively.

#### The division rate of T lymphocytes is not significantly affected by irradiation

To further investigate the proliferative characteristics of T lymphocytes and the subsequent effects of X-ray irradiation, we determined the lymphocyte proliferation index (LPI) for each irradiation dose, for all time points. This index determines the average number of divisions made by all actively proliferating cells and thus reflects the division rate of the PHA-responsive T lymphocytes.

After 72 h of culture, non-irradiated T lymphocytes completed an average of 1.59 divisions, whereas the 1 and 2 Gy irradiated cells divided 1.55 and 1.61 times on average, respectively (Supplementary Fig. S6 and Table S2). An average of 2.92, 2.70, and 2.68 divisions were performed after 96 h of culture, for 0, 1, and 2 Gy, respectively. Again, no significant differences were observed between the irradiation doses (*p* > 0.05), indicating no profound effect of irradiation on the subsequent division rate of T lymphocytes. Markedly, our results demonstrate an average division rate of more than one division in 24 h, suggesting that the duration of the T lymphocyte cell cycle is shorter than this period of time (Supplementary Table S2).

### Tracking the cell cycle progression of T lymphocytes in whole blood cultures using EdU pulse-chase analysis

#### The EdU pulse-chase method was successfully optimized for T lymphocytes cultured in whole blood

**Figure 3:**
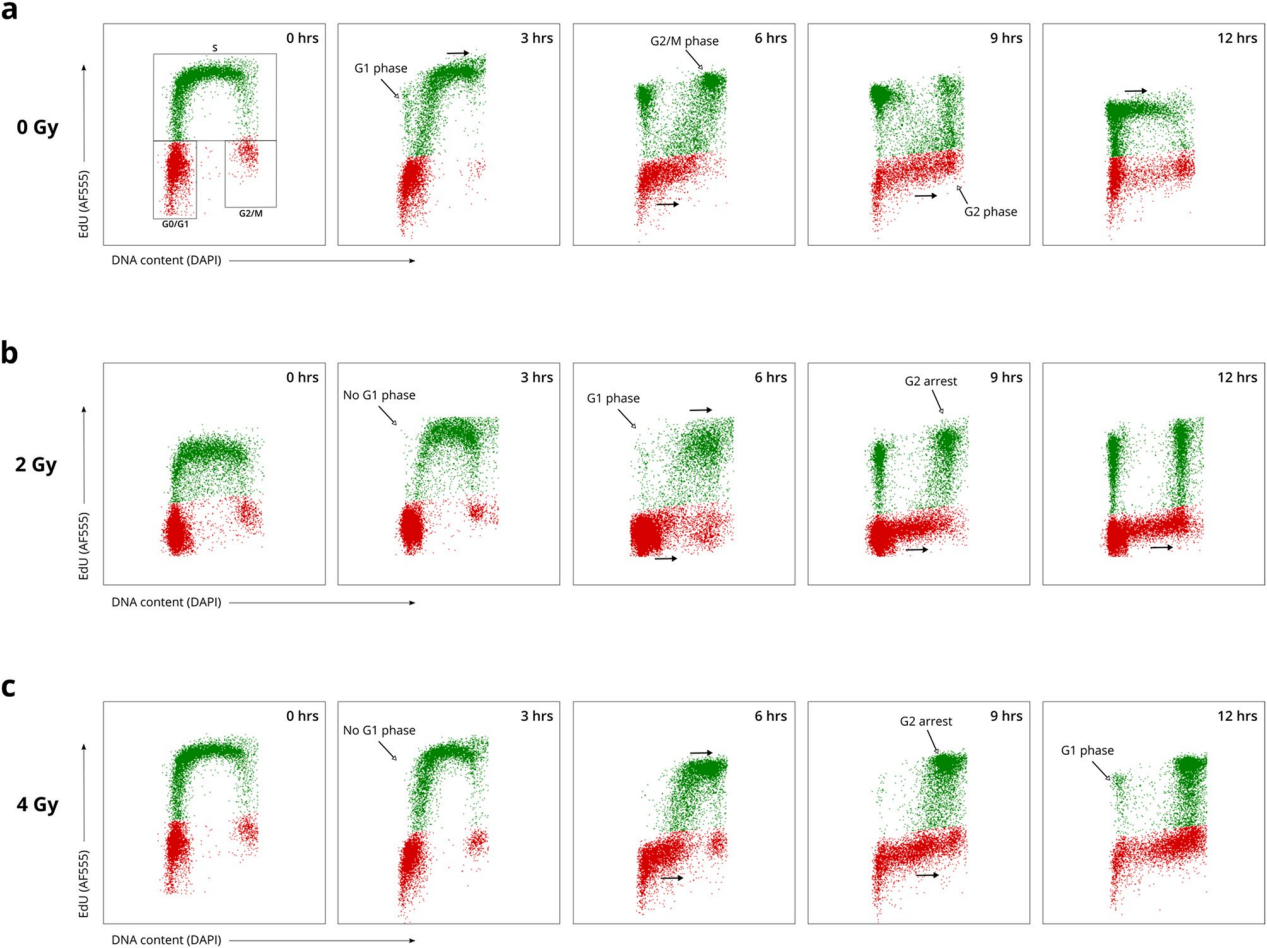
EdU pulse-chasing of T lymphocytes in whole blood cultures allows for the assessment of the cell cycle progression. T lymphocyte proliferation was stimulated with PHA for 96 h before EdU pulse-labeling. (**a**) Bivariate distributions of non-irradiated EdU pulse-labeled T lymphocytes showing DNA content (x-axis) and EdU incorporation (y-axis). The green population shows the EdU-positive T lymphocytes. The red population shows the EdU-negative cells. The black arrows indicate the progression of the cells through the cell cycle, over time. The bivariate profiles of one donor are displayed here. Other donors show similar distributions. (**b**) Bivariate profiles of T lymphocytes exposed to 2 Gy of X-rays, visualized as described for (a). (**c**) Bivariate profiles of T lymphocytes exposed to 4 Gy of X-rays, visualized as described for (a).

We successfully adapted the EdU pulse-chase method for T lymphocytes cultured in whole blood. Firstly, no effect of EdU pulse-labeling on the cell cycle distribution of both non-irradiated and irradiated T lymphocytes was observed, showing the applicability of this method (Supplementary Fig. S3). Secondly, EdU-labeled S phase cells can clearly be identified (green cohort) and a distinction between all cell cycle phases can be made through bivariate analysis of EdU incorporation and cellular DNA content (Fig. 3a, 0 h). This demonstrates the effectiveness of our technique to monitor T lymphocyte cell cycle progression in whole blood cultures. Thirdly, our adapted technique was also successful for chasing EdU-labeled T lymphocytes over time (Fig. 3a). The shifting patterns of the bivariate profiles show the T lymphocytes progression through the cell cycle. Over time, we can see the EdU-labeled cohort moving from the S to the G2 phase (Fig. 3a 3–6 h). As the cells divide, the cells will progress back to the G1 phase, thus starting a new cell cycle (Fig. 3a, 3–9 h). Continuous proliferation can be seen as the EdU-labeled cells progress to the S phase of a second cycle (Fig. 3a, 12 h). Progression of the unlabeled G1 and G2/M phase cells can also be observed (Fig. 3a, red cohort). Lastly, our results show that this technique can be used in combination with irradiation of the cells, which allows for the evaluation of the effects of irradiation on the cell cycle progression (Fig. 3a–c).

#### Quantitative analysis of the cell cycle phase durations of non-irradiated T lymphocytes

By employing multiple quantitative analyses on both the EdU pulse-chase and CFSE proliferation data, the cell cycle kinetics of non-irradiated T lymphocytes were examined more in detail. These methods allow us to estimate the duration of the cell cycle phases and to define the total cell cycle time (Tc).

**Figure 4:**
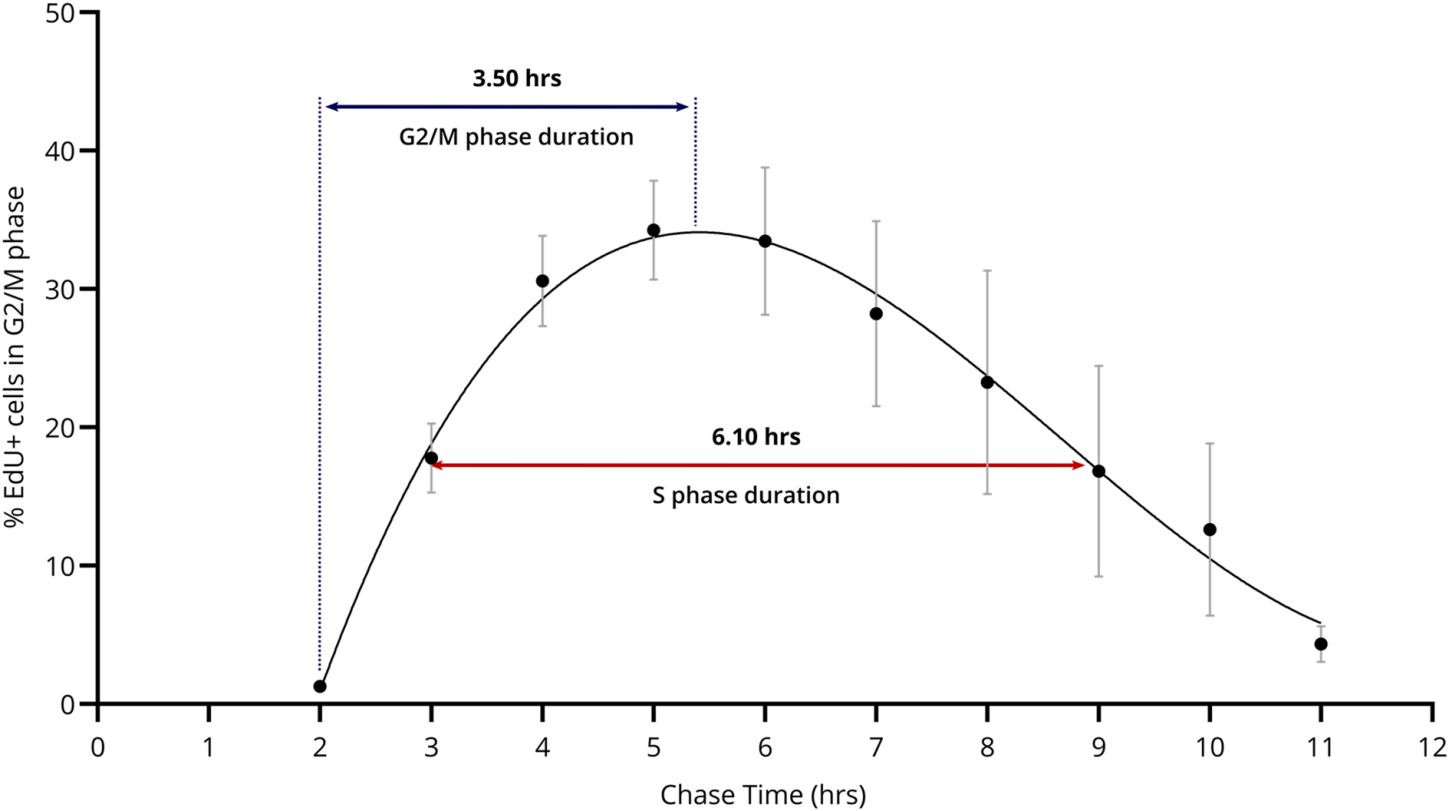
Estimation of the S and G2/M phase duration through the quantification of non-irradiated EdU-labeled cells in G2/M phase over time. Datapoints show the mean percentage of non-irradiated EdU-positive cells in the G2/M phase across four independent experiments, for each time point. Error bars show the standard deviation of the mean. A cubic fit was applied, on which the full-width-at-half-maximum was calculated, as shown by the red arrow. The blue arrow indicates the G2/M phase duration.

The duration of both the S and the G2/M phase was estimated by quantifying the progression of EdU-labeled cells through the G2/M phase (Fig. 4; Table 1). Importantly, we can see that the estimated S phase length (6.10 ± 1.38 h) and G2/M phase length (3.50 ± 0.69 h) are consistent with the T lymphocyte progression visible in the bivariate profiles (Fig. 3a; Supplementary Fig. S7). For instance, we can see the first EdU-labeled cells appear in the G1 phase around 3 h after labeling, which matches the estimated G2/M phase duration (Fig. 3a; 3 h profile, indicated by the arrow). Additionally, verification of the estimated S phase duration with the relative movement (RM) technique showed a similar outcome (6.75 ± 0.83) (Table 1; Supplementary Fig. S4).

**Table 1:** The estimated duration (h) of each cell cycle phase and total cell cycle time (Tc) of non-irradiated T lymphocytes, determined by different methodologies.

Phase	G1	S	G2/M	Tc
CFSE proliferation				16.62 ± 1.84
EdU pulse-chase		6.10 ± 1.38	3.50 ± 0.69	
RM Technique		6.75 ± 0.83		
Derived from Tc, S, and G2/M	7.02			

The estimated mean ± standard deviation (h) is shown.

Due to increasing asynchrony after the first cell cycle, the G1 phase duration is more difficult to directly quantify from the EdU pulse-chase results. We therefore derived an estimate of the total cell cycle duration (Tc) from the CFSE-based lymphocyte proliferation index, as described above, which resulted in an estimated Tc of 16.62 ± 1.84 h (Supplementary Table S1). By subtracting the S and G2/M phase lengths from this Tc value, the average G1 phase duration was estimated to be 7.02 h (Table 1). Based on the standard deviations of the S, G2/M, and Tc durations, we expect variation on this estimated value. This is also reflected in the bivariate profiles (Fig. 3a; Supplementary Fig. S7).

#### Quantitative and qualitative analysis of the effects of irradiation on the cell cycle progression of T lymphocytes

The influence of X-ray irradiation on the cell cycle progression was assessed using both quantitative and qualitative methods. T lymphocytes were exposed to 2 and 4 Gy of irradiation during EdU pulse-labeling and the cells were chased over time. To determine whether irradiation has an impact on the duration of the S phase, we used the RM technique. Our results show no significant effect of 2 Gy of X-rays on the S phase length (7.33 ± 0.58 h; *p* > 0.05) compared to 0 Gy (6.75 ± 0.96 h) (Supplementary Fig. S4). However, when exposed to 4 Gy of irradiation, the EdU-labeled cells show a significant prolongation of the S phase duration (9.33 ± 0.58 h; *p* < 0.05) compared to non-irradiated cells.

**Figure 5:**
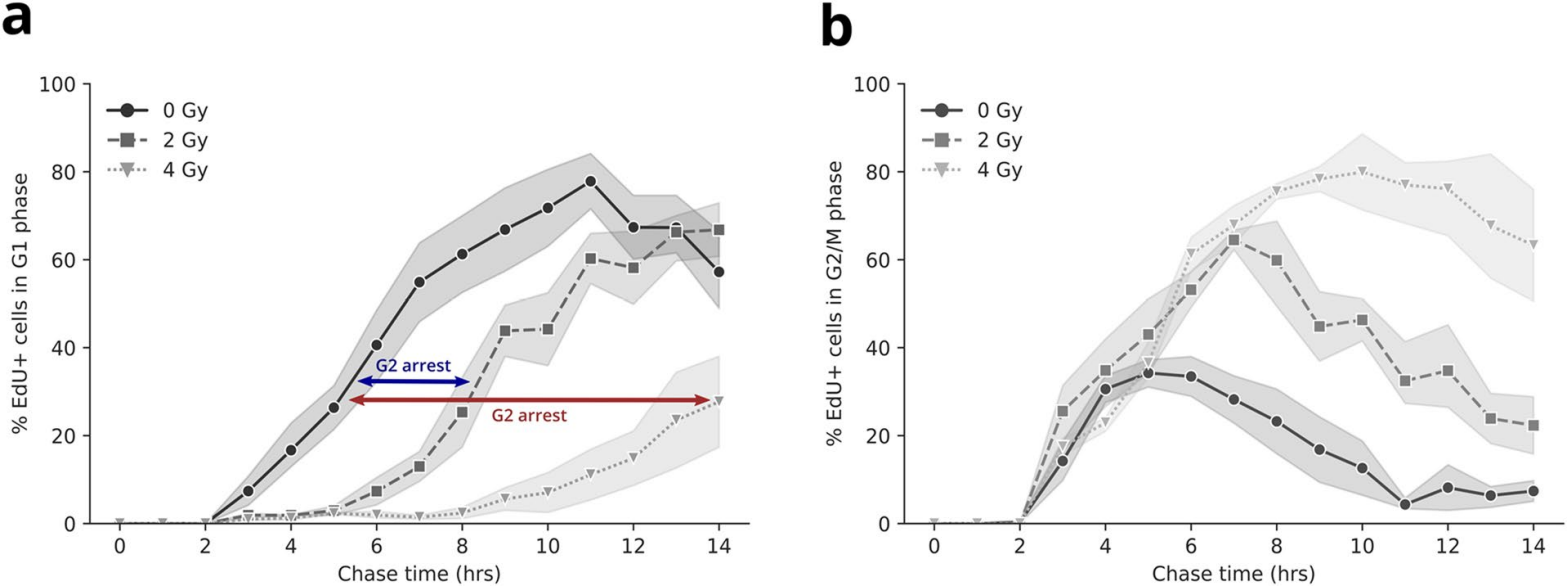
Progression of EdU-labeled T lymphocytes through both the G1 and the G2/M phase over time. (**a**) Datapoints show the mean percentage of EdU-positive cells for each irradiated dose in the G1 phase, for all time points. The grey shading shows the standard deviation of three independent experiments. The blue and red arrow indicate the cell cycle delay for 2 and 4 Gy, respectively. (**b**) The mean percentage of EdU-positive cells in the G2/M phase is shown for each irradiation dose, for each time point. The grey shading shows the standard deviation of three independent experiments.

Further quantifications of the cell cycle phase durations were complicated by considerable variation in the samples due to an increase in asynchrony of the dividing cells. We therefore focused on the qualitative properties of the progression through both the G2/M and the G1 phase of the EdU-labeled cells and the differences between irradiated and non-irradiated cells. When examining the G2/M-to-G1 phase progression of EdU-labeled cells, a delay of re-entry in the G1 phase can be observed for the irradiated cells (Figs. 3b–c, 5a). This delay corresponds to a prolonged G2/M phase, indicating considerable G2 arrest after irradiation (Fig. 5b; Supplementary Figs. S8, S9). Our data show that after exposure to 2 and 4 Gy of X-rays, the peak in number of cells in G2/M phase is reached on average 2 and 5 h later, respectively, compared to the non-irradiated (Fig. 5b). Interestingly, when observing the entry of EdU-labeled cells into the next G1 phase and the correlated G1-entry kinetics, no major differences can be observed between the T lymphocytes exposed to 0 versus 2 Gy of X-rays, besides the delay of G1-entry (Fig. 5a). When examining the cells exposed to 4 Gy of X-rays, however, a flattening of the curve is visible, indicating that a large proportion of these cells are unable to complete their cell cycle over the time course analyzed in this study.

#### T lymphocytes remain in G2 arrest up to 25 h after irradiation

**Figure 6:**
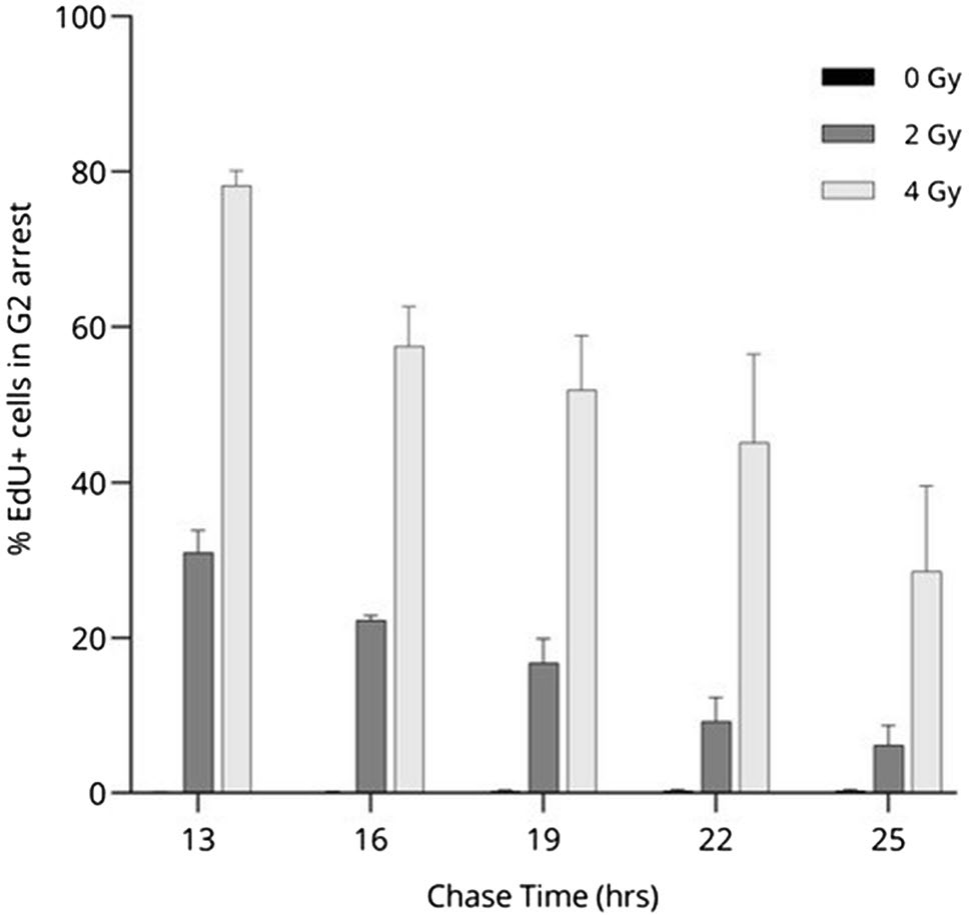
G2 arrest of EdU-labeled T lymphocytes after exposure to X-ray irradiation over time. The bar plots show the mean percentage of EdU-positive cells still residing in G2 arrest for non-irradiated and irradiated conditions, over multiple time points. Error bars show the standard deviation of the mean of three independent experiments.

As previous results showed that T lymphocyte cell cycle progression is delayed in the G2/M phase up to 14 h after irradiation in the S phase, we further examined the extent of G2 arrest by tracking the EdU-labeled cells for 25 h, with 3 h-intervals (Fig. 6). We observed a substantial percentage of cells arrested in the G2 phase 13 h after 2 Gy and 4 Gy of irradiation. This percentage decreased over time, as cells eventually progressed to the G1 phase. Markedly, 25 h after irradiation, 6.14% and 28.56% of EdUlabeled T lymphocytes still resided in the G2 phase for 2 and 4 Gy respectively, showing a prolonged arrest for a minority of cells irradiated in the S phase.

#### T lymphocytes irradiated in the G1 phase do not undergo G1 arrest

We also examined the progression of the EdU-negative T lymphocytes through the cell cycle, visible in the bivariate profiles as the red cohort (Fig. 3a–c, Supplementary Fig. S7–S9) and compared the distributions of the irradiated and non-irradiated samples. We observed that cells irradiated in the G1 phase displayed no pronounced delay of entry into the S phase, indicating no major G1 arrest after exposure up to 4 Gy of X-rays. Instead, an accumulation of the non-labeled irradiated cells in the G2/M phase could be seen, suggesting that these cells do experience a G2 arrest.

## Discussion

In this study, we report on the proliferative characteristics of T lymphocytes after stimulation with the mitogen phytohaemagglutinin (PHA) and, additionally, we demonstrated the impact of X-ray irradiation on the division capability of these cells. Our results show that a first division, after stimulation with PHA, is completed between 48 and 72 h. Interestingly, exposure to X-ray doses up to 2 Gy before the proliferative stimulus did not significantly alter this timespan. A marked asynchrony in the proliferating population could be observed for both the irradiated and non-irradiated T lymphocytes, which is in concordance with previous reports^13,14^. This asynchrony can be attributed to the fact that the time span required for a first division is significantly longer than the time spans for subsequent divisions, thus resulting in a heterogeneously dividing population.

Strikingly, we observed that a significant proportion of T lymphocytes did not respond to the proliferative stimulus, even after 96 h of culture. On average, only 50% of the original T lymphocytes — indicated by the precursor frequency — responded to the PHA stimulus, with some proliferative responses being as low as ~ 37%. This is in contrast to previous reports dating from the late 1970s, where a proliferative response to PHA stimulus was reported for more than 80% of the cells^3,4^. However, the methodologies used in previous reports did not take into account the continuous proliferation of the cells, and thus the exponentially expanding population. This demonstrates the importance of a good metric, such as the precursor frequency, which describes the initial proliferative response of the cells, without being biased by any potential differences in the division rate.

It is important to note that the determined PF could be a minor underestimation due to a potential limited presence of other lymphocytes in the sample, namely B lymphocytes and NK cells^27^. The PF is determined based on the complete cell population, including any potential non-proliferating cells. As no extra T lymphocyte-specific staining was included in the gating strategy, this minor fraction of cells could introduce a small bias into our results. Nevertheless, based on previous reports, only a small fraction of these cells (< 10%) is estimated to be present in the samples. Therefore, this bias is expected to be relatively minor, and should not impact the overall results and conclusions.

Our results showed no significant impact of irradiation on both the proportion of dividing cells and the subsequent division rate of the proliferating cells. These findings suggest that the cells did not undergo cell cycle arrest after exposure to 1 and 2 Gy of X-rays in the G0 phase, as this would have resulted in a decreased proportion of dividing cells due to arrest at the cell cycle checkpoints^34^. This indicates that most of the DNA damage induced by irradiation was repaired before continuing their cell cycle progression, which is not unexpected as it is known that the main repair pathway active in the G0 phase, namely the non-homologous end joining (NHEJ) pathway, ensures repair of DNA damage within 24 h^35,36^. It is important to note that in these experiments, the cells were exposed to moderate doses of X-rays and that higher doses (> 7.5 Gy) have been reported to inhibit the proliferative response of lymphocytes, as shown by multiple studies^37,38^. Furthermore, it is also important to note that the whole lymphocyte fraction was analyzed assuming only T lymphocyte proliferation, as it widely known that PHA as mitogen has a stimulating effect specifically on T lymphocytes^25–27,39^. A minor fraction of other lymphocytes, namely B lymphocytes and NK cells, can still remain present in the culture. However, these cells do not initiate proliferation after PHA addition. Importantly, as all aspects of this research only considered proliferating cells, with exception of the precursor frequency, no influence on the results of this minor fraction of cells is expected. In future experiments, an additional staining for CD3 + could prove to be of value, especially to determine the precursor frequency more accurately as we expect this to be a small underestimation of the actual T lymphocyte PF.

Various studies have mentioned the potential adverse effects of CFSE on lymphocytes^40,41^. These studies demonstrated a considerable effect on cell viability and proliferative capacity of high concentrations of CFSE, ranging from 5 to 10 µM. Labeling concentrations of 2.5 µM or lower, however, resulted in only a slightly diminished cell viability. Based on these reports, we expect no considerable negative effect of CFSE labeling on the proliferative capability of the cells used in our experiments.

Proliferation assays, using e.g. CFSE, are excellent methods to investigate the proliferative characteristics of lymphocytes, however, they provide us with only limited information on the progression of these cells through the cell cycle and the corresponding kinetics. A method that forms a particularly powerful approach for monitoring cell cycle progression is based on DNA labeling of proliferating cells with a thymidine nucleoside-analogue, usually BrdU or EdU^42,43^. Combining this technique with traditional cell cycle analysis allows for an efficient flow cytometry-based assessment of the cell cycle distributions^44^. In our study, we successfully optimized the EdU pulse-chase method for tracking the cell cycle progression of PHA-stimulated T lymphocytes cultured in whole blood. Beside its advantages of being easy, fast, and accurate, it also provides us with a model that better represents the in vivo situation^45,46^. A disadvantage reported in literature, however, is the potential cytotoxic effect of EdU associated with strong cell cycle perturbations and even cell death^43,47–49^. This detrimental effect is reported to be cell type dependent and has been linked with the used EdU labeling concentration and labeling time. Importantly, our results demonstrate no impact of EdU pulse-labeling on the cell cycle progression of the T lymphocytes, indicating no immediate cytotoxic effect of EdU. Furthermore, we observed that irradiating the cells during pulse-labeling had no adverse effect on the functionality of this method.

Using this technique, we were able to make an estimation of each cell cycle phase duration and simultaneously analyze the effects of irradiation on the different cell cycle phases. We estimated the cell cycle phase (G1, S, and G2/M) durations to be 7, 6.1, and 3.5 h, respectively. The total cell cycle time was estimated to be 16.6 h. To our knowledge, this is the first study to determine the T lymphocyte cell cycle kinetics with more up-to-date techniques since the ‘90s.

We observed a prolongated G2 phase of cells irradiated in the S phase, indicating that the cells are halted at the G2/M cell cycle checkpoint. The extent of the observed delay was dependent on the delivered dose, as demonstrated by chasing the cells through prolonged culture times. This observation is in agreement with previous reports^50,51^. Markedly, our results show similar kinetics for G1 phase-entry between non-irradiated cells and cells exposed to 2 Gy of X-rays, although there is a ~ 2 h-delay. In contrast, strong cell cycle disturbances could be observed when exposed to 4 Gy of X-rays, with deviating S and G1 phase kinetics and ~ 30% of the cells still halted in G2/M phase up to 25 h after irradiation.

When observing cells irradiated in the G1 phase, we did not see a delay of S phase-entry, indicating that these cells do not experience a pronounced G1 arrest, even after exposure to 4 Gy of X-rays. This was a surprising observation, as it has been suggested in other cell types that the G1-S checkpoint is a master regulator that prevents cell cycle progression of damaged cells^34,52^.

Various other techniques are available to study cell cycle kinetics, such as the FUCCI system^53^ or the fraction-labelled-mitoses (FLM) assay^54^. However, compared to these techniques, the EdU pulse-chase assay is still a relatively cost-efficient and simple method and, as our results demonstrate, this assay can be performed on whole blood samples. We do note that, in contrast to S and G2/M phase, the duration of the G1 phase was difficult to quantify from these EdU pulse-chase experiments. We therefore derived the G1 phase length by subtracting the S and G2/M phase durations from the estimated total cell cycle time (Tc) determined by the CFSE experiments. Importantly, these findings are in concordance with visual examination of the EdU-positive cohort progression through the G1 phase in the bivariate profiles. Additionally, this technique does not allow subdivision between the G2 and M phase, which impedes determination of the duration of these phases separately.

In recent years, considerable progress has been made towards understanding the precise mechanisms involved in cell cycle progression, checkpoints, and DNA repair^50,53,55^. However, the cell cycle is regulated by a complex network and is influenced not only by intracellular signals, but also by extracellular factors. Nonetheless, elucidating the processes that are the foundation of T lymphocyte cell cycle progression and their unique responses to genotoxic agents such as X-rays is essential in many medical and biological research fields. It can aid in the screening of potential new drugs that can selectively modulate the cell cycle transition or assist in the development of novel treatment modalities such as cell cycle regulators, that are considered attractive targets in cancer therapy^56–58^. Moreover, further investigation into the cell cycle profiles of the T lymphocyte subsets, such as CD4 + and CD8 + T lymphocytes or T lymphocytes expressing either αβ or γδ T cell receptors (TCR), may be of interest for future research as it could reveal certain fundamental differences that could aid in the development of new therapies^59–61^. Furthermore, various processes modulating the cell cycle regulatory system are also closely linked with the inhibition or induction of cell death mechanisms^62^. Exposure to ionizing radiation has been known to elicit cell death through various processes, such as apoptosis, mitotic catastrophe, necroptosis, or senescence^63^. Not surprisingly, studies into cancer therapy development are focused on understanding these mechanisms, and further research into the effects of irradiation on the cell cycle regulatory system, and associated cell death processes, can prove to be of therapeutic interest^64–66^.

In conclusion, the use of both the CFSE proliferation assay and EdU pulse-chase assay allowed us to visualize the cell cycle kinetics of human lymphocytes and the correlated effects after irradiation. Importantly, by using both assays, we were able to visualize and quantify the lymphocyte proliferation characteristics starting from first division to fully dividing cells. Studying the impact of genotoxic agents, such as irradiation, on cell cycle behavior is crucial for our ability to understand and predict cellular responses.

## Supplementary Information

Supplementary Information.

## Data Availability

The datasets generated during and/or analyzed during the current study will be made available.
