# Supplementary material for: An updated view into the cell cycle kinetics of human T lymphocytes and the impact of irradiation
Source: Sci Rep. 2022 May 10;12:7687. doi: 10.1038/s41598-022-11364-9 (PMC9090834; doi:10.1038/s41598-022-11364-9)

An updated view into the cell cycle kinetics of human T lymphocytes and the impact of irradiation

Evi Duthoo^1,2^, Anne Vral^1,2^, Ans Baeyens^*1,2^

^1^ Radiobiology Group, Department of Human Structure and Repair, Ghent University, Ghent, Belgium
^2^ Cancer Research Institute Ghent (CRIG), Ghent, Belgium

# Supplementary Figures and Tables

## Supplementary Figures

*
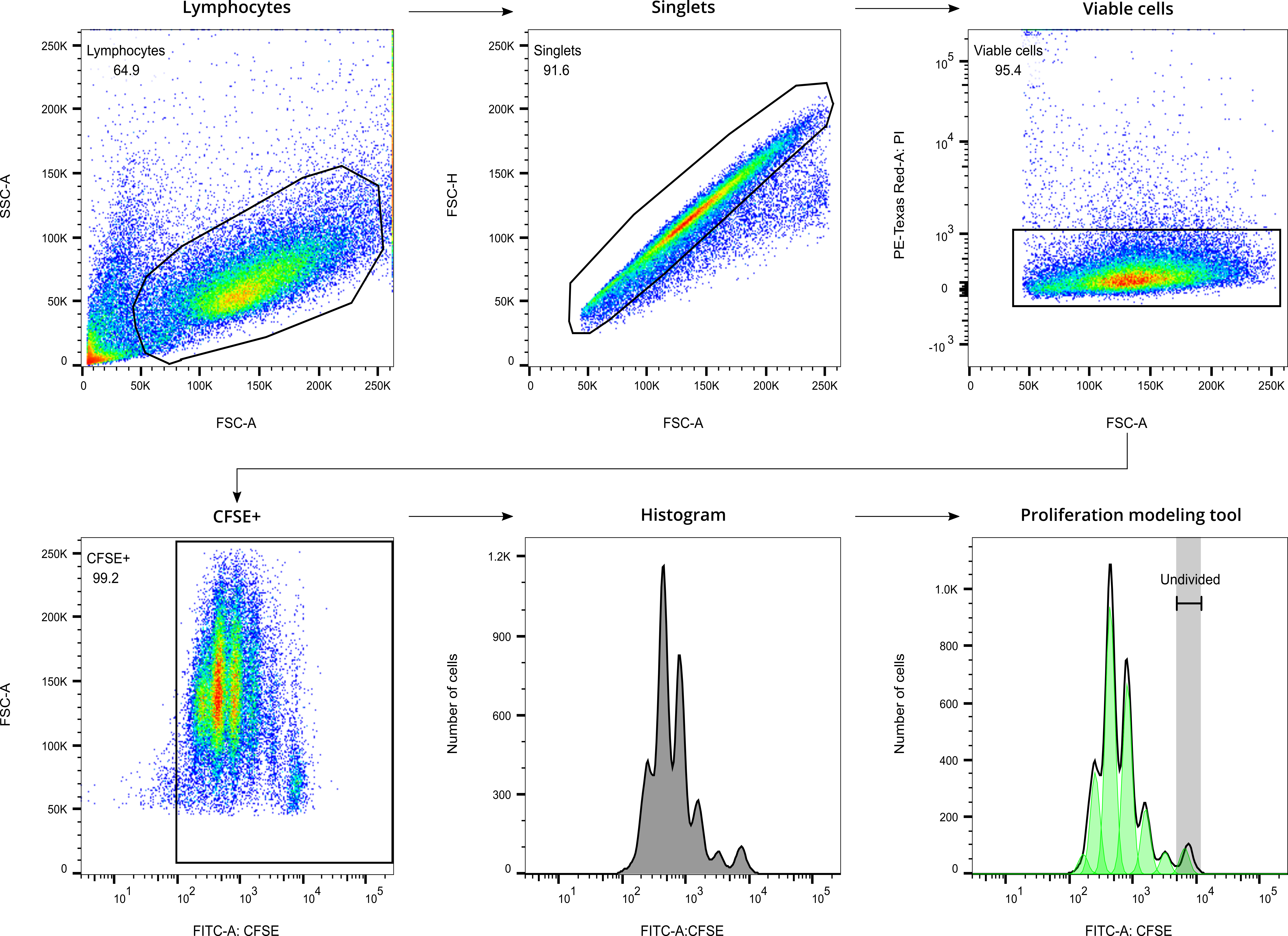
*

**Supplementary Figure S1. Flow cytometric gating strategy for CFSE proliferation sample analysis.** Data was analyzed using FlowJo software. Lymphocytes were identified based on forward scatter (FSC) and side scatter (SSC). Doubles were excluded using FSC-A and FSC-H. Viable cells were selected based on propidium iodide (PI) staining. CFSE positive T lymphocytes were gated and autofluorescence cells were excluded. The CFSE positive cells were analyzed using the Proliferation Modeling Tool available in FlowJo Software package.

*
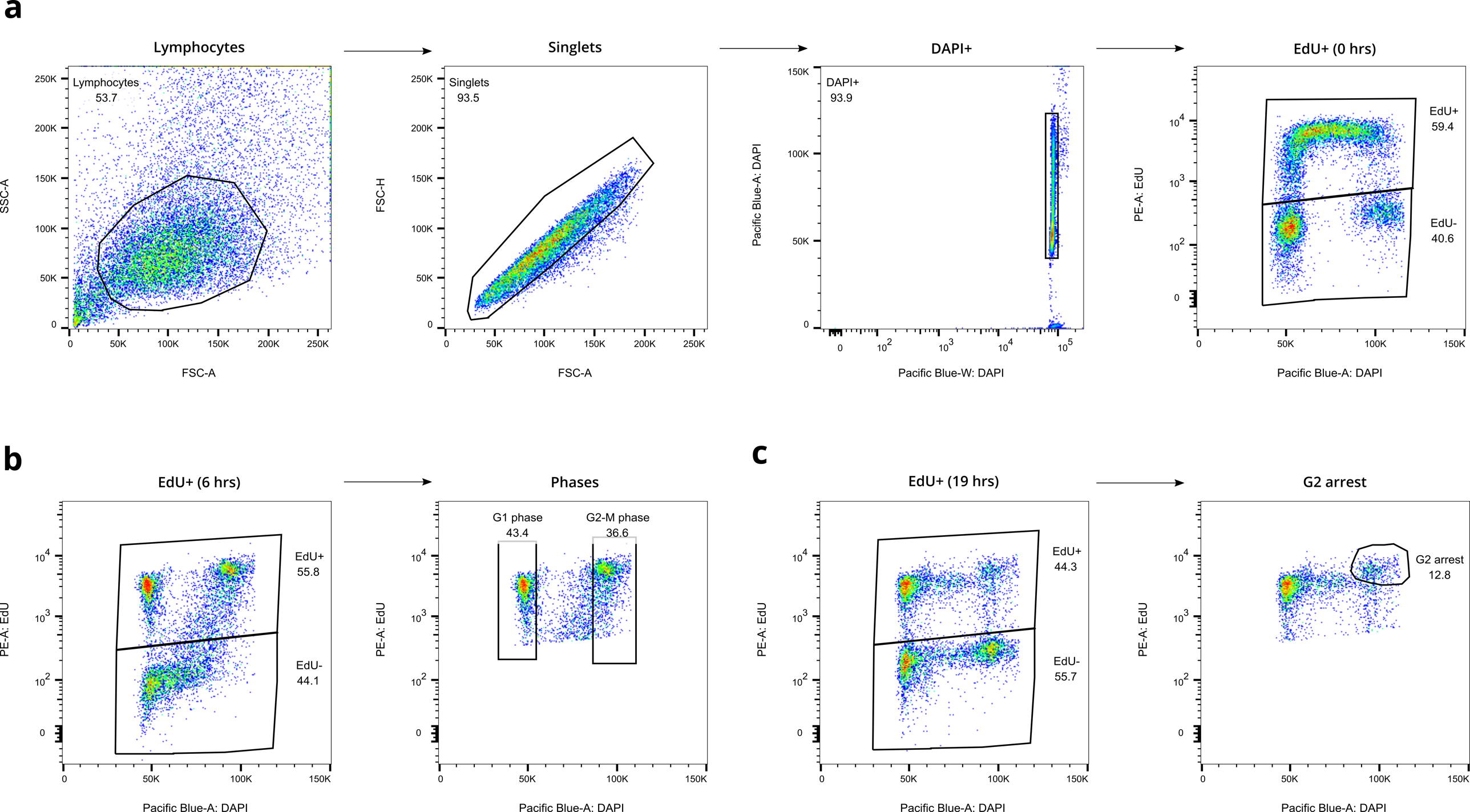
*

Supplementary Figure S2. Flow cytometric gating strategy for EdU pulse-chase sample analysis. Data was analyzed using FlowJo software. (a) Lymphocytes were identified based on forward scatter (FSC) and side scatter (SSC). Doubles were excluded using FSC-A and FSC-H. DAPI positive cells were selected and subsequently, EdU positive T lymphocytes were identified. (b) Analysis as described in (a). Additional gatings to determine the fraction of G1 and G2/M phase cells. (c) Analysis as described in (a). Additional gating to determine the fraction of cells in G2 arrest.


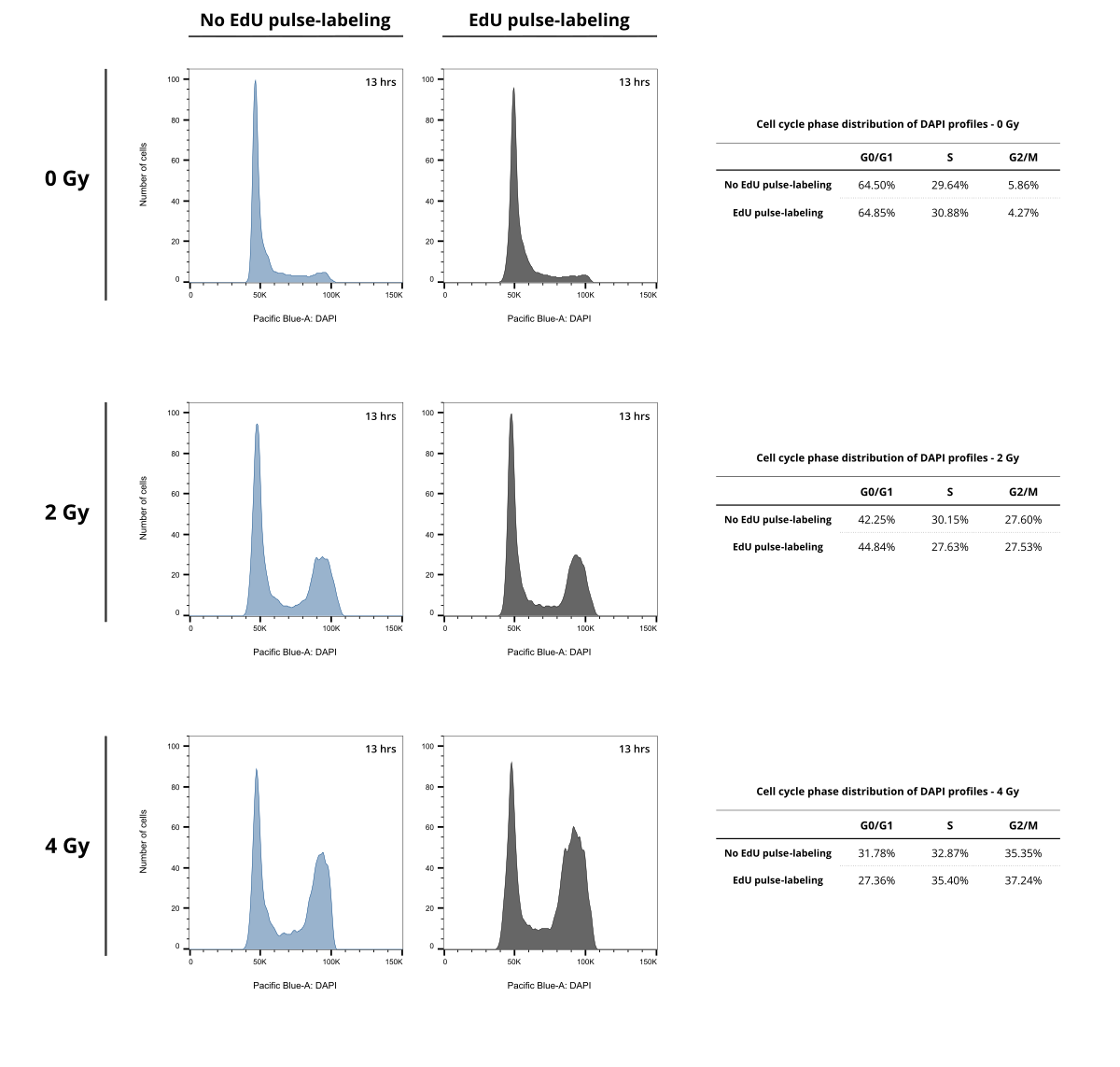


**Supplementary Figure S3. Effect of EdU pulse-labeling on the cell cycle profiles of irradiated and non-irradiated T lymphocytes after 13 hrs.** T lymphocyte cell cycle profiles (DAPI) of both EdU pulse-labeled (10 µM, 30 min) (grey profile) and non-labeled (blue profile) whole blood cultures, 13 hrs after exposure to 0, 2, and 4 Gy of X-rays. The DAPI profiles were analyzed using the Cell Cycle Analysis tool available in the FlowJo Software package to quantify the cell cycle phase distributions.


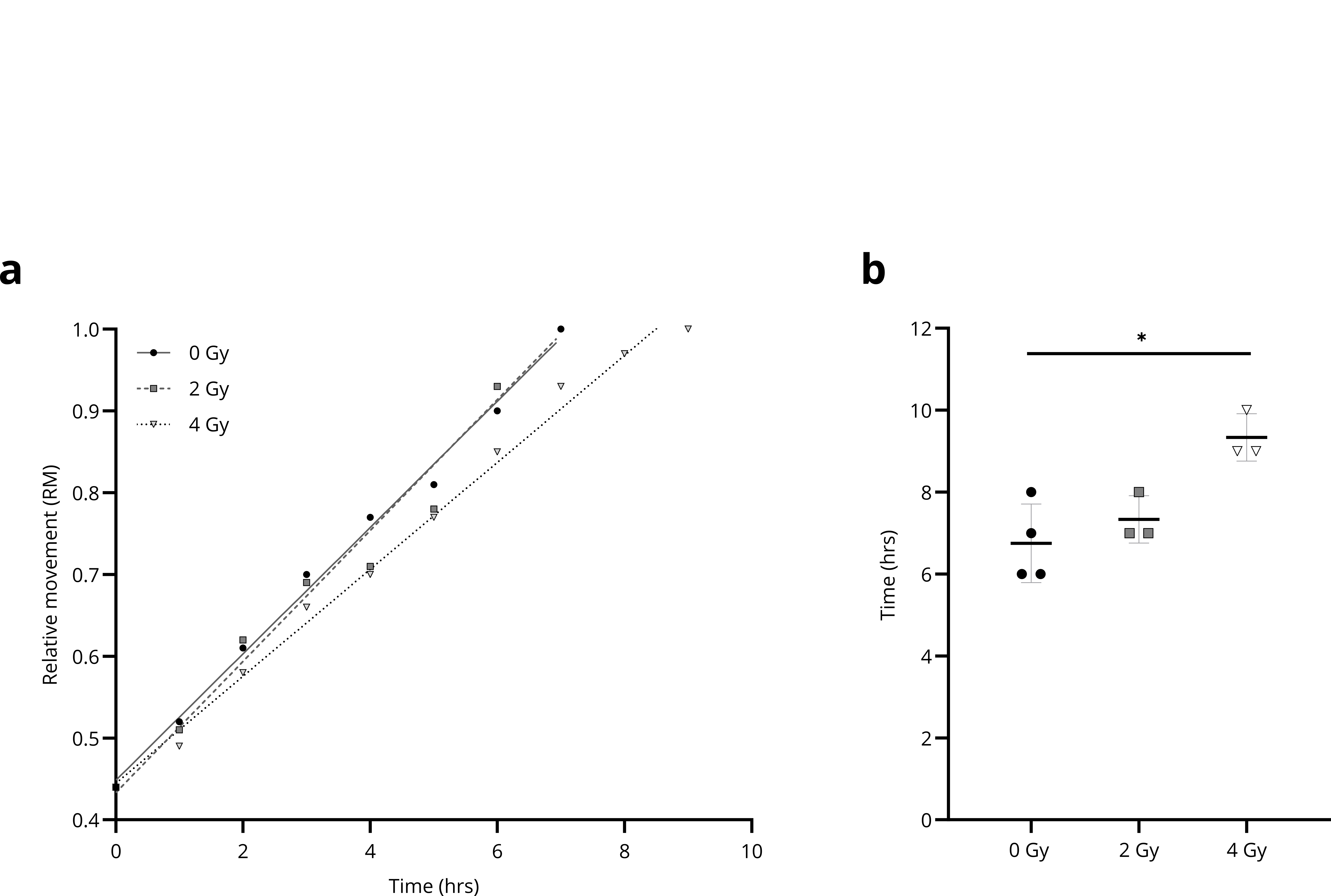


**Supplementary Figure  S4. The relative movement (RM) technique is used to determine the duration of the S phase.** As described by Begg et al (1985), the relative movement (RM) method can be used to analyze the progression of EdU-labeled cells through the S phase, relative to the G0/G1 and G2/M cohort. If the cells are uniformly distributed throughout the S phase, the RM will equal 0.5. When the EdU-labeled cells start progressing from the S to G2 phase, the RM increases. Eventually, when the EdU-labeled cells reach the G2 phase – and have thus completed the S phase – the RM will equal 1. This time point marks the duration of the S phase. (a) An example of the RM of non-irradiated and irradiated T lymphocytes (y-axis) over time (x-axis) is shown. As the RM reaches 1, the corresponding time point indicates the duration of the S phase. (b) An estimation of the duration of the S phase for 4 independent experiments based on the RM technique. Non-irradiated T lymphocytes (black circles, 0 Gy) show an average duration of 6.75 ± 0.96 hrs. T lymphocytes exposed to 2 Gy (dark grey squares) and 4 Gy (white triangles) of X-rays have an average S phase duration of 7.33 ± 0.58 hrs and 9.33 ± 0.58 hrs, respectively. Error bars indicate the standard deviation. *, p<0.05 (p = 0.0388)


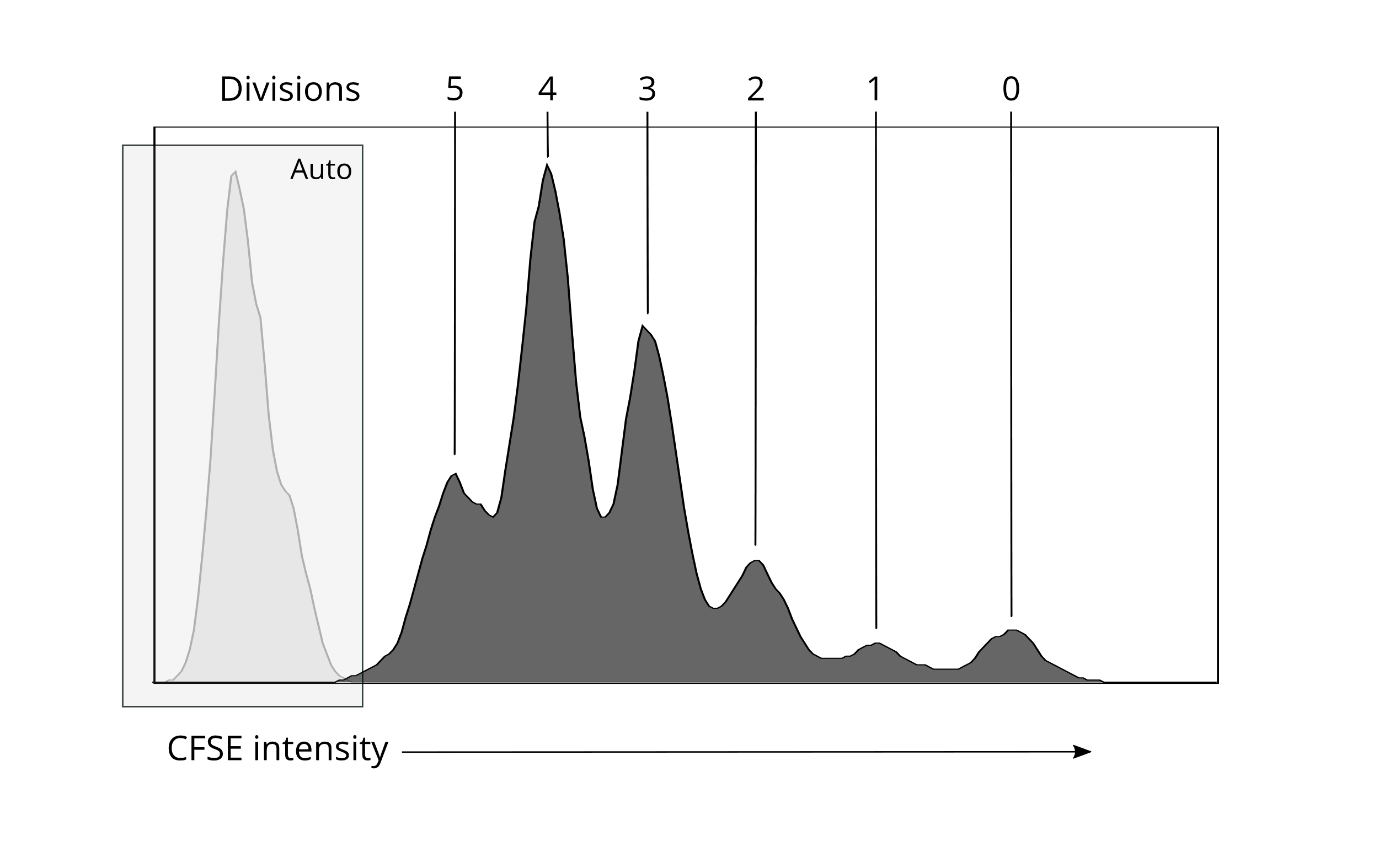


**Supplementary Figure S5.** **CFSE profile of isolated PHA-stimulated T lymphocytes.** The dark grey histogram shows that proliferation of CFSE-labeled cells results in the sequential halving of the fluorescence signal with each division. The traceable number of divisions is displayed above the histogram. The light grey histogram shows the autofluorescence profile of the stimulated non-labeled control sample.

**

**Supplementary Figure S6.** **Effect of irradiation on the lymphocyte proliferation index (LPI) of PHA-stimulated T lymphocytes.** CFSE-labeled T lymphocytes were irradiated with 0, 1, and 2 Gy of 220 kV X-rays and subsequently cultured in the presence of PHA for 24, 48, 72, and 96 hrs. The lymphocyte proliferation index (LPI) was determined for each irradiation dose and is plotted for every time point. Error bars show the standard deviation on the mean of 6 independent experiments. For samples where none of the cells have started proliferating, the LPI cannot be calculated, hence not all samples are included in the figure.

*
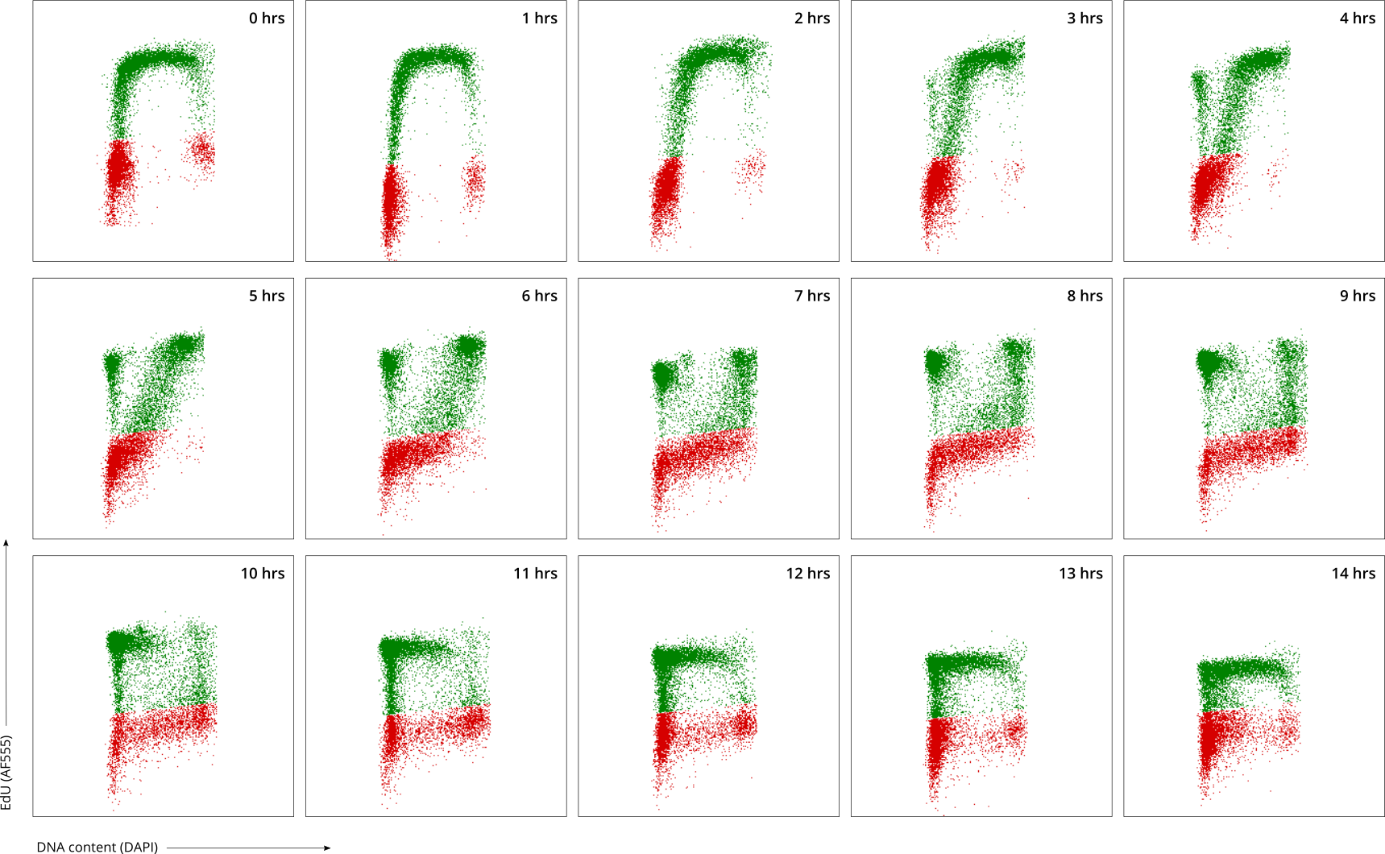
*

Supplementary Figure S7. EdU pulse-chasing of non-irradiated T lymphocytes over time. Bivariate distributions of EdU pulse-labeled T lymphocytes indicated by DNA content (x-axis) and EdU incorporation (y-axis). The green population shows the EdU-positive T lymphocytes. The red population shows the EdU-negative cells. The bivariate profiles of one sample are displayed here. Other samples show similar distributions.

*
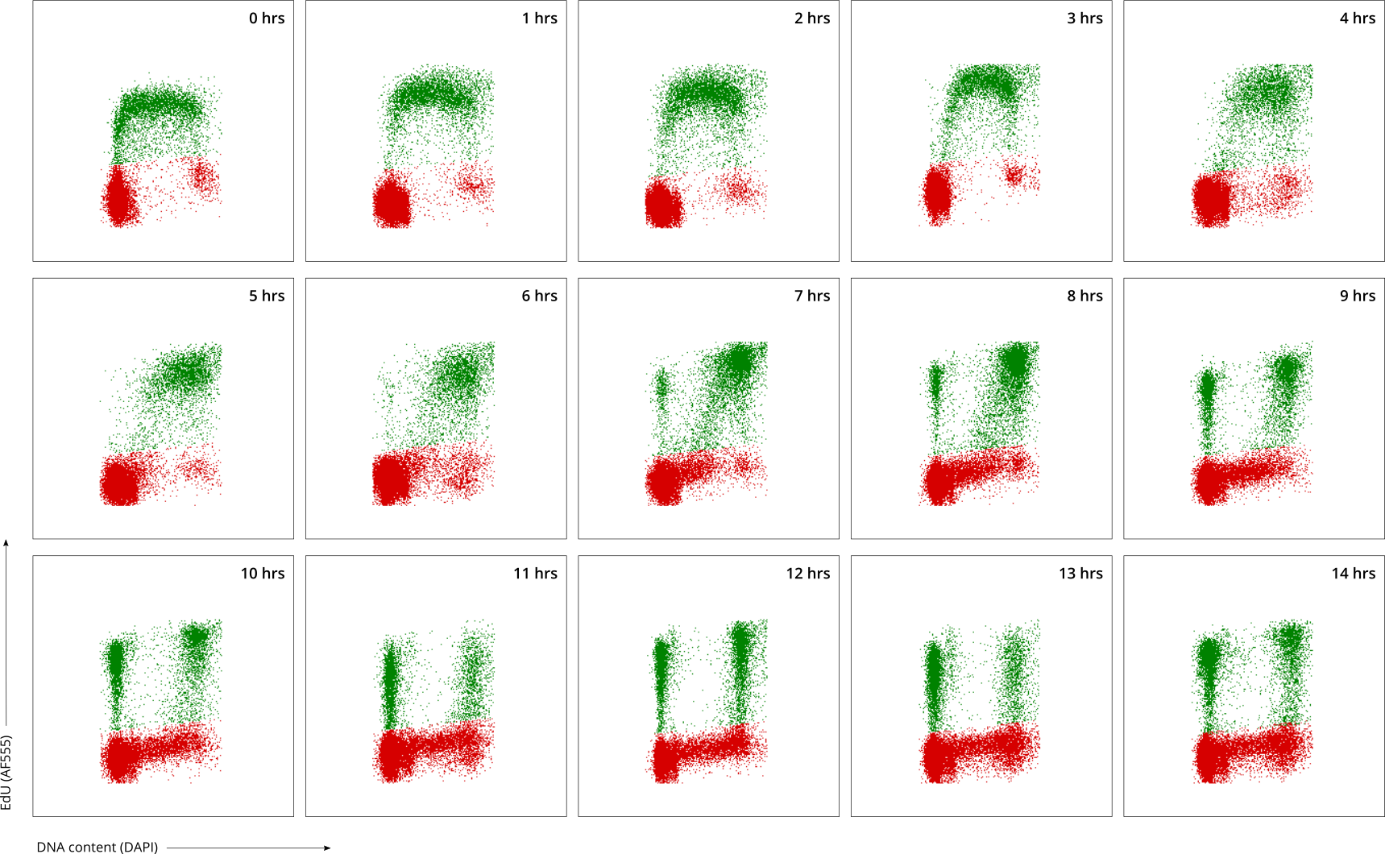
*

Supplementary Figure S8. EdU pulse-chasing of T lymphocytes over time after exposed to 2 Gy of X-rays. Bivariate distributions of EdU pulse-labeled T lymphocytes indicated by DNA content (x-axis) and EdU incorporation (y-axis). The green population shows the EdU-positive T lymphocytes. The red population shows the EdU-negative cells. The bivariate profiles of one sample are displayed here. Other samples show similar distributions.

*
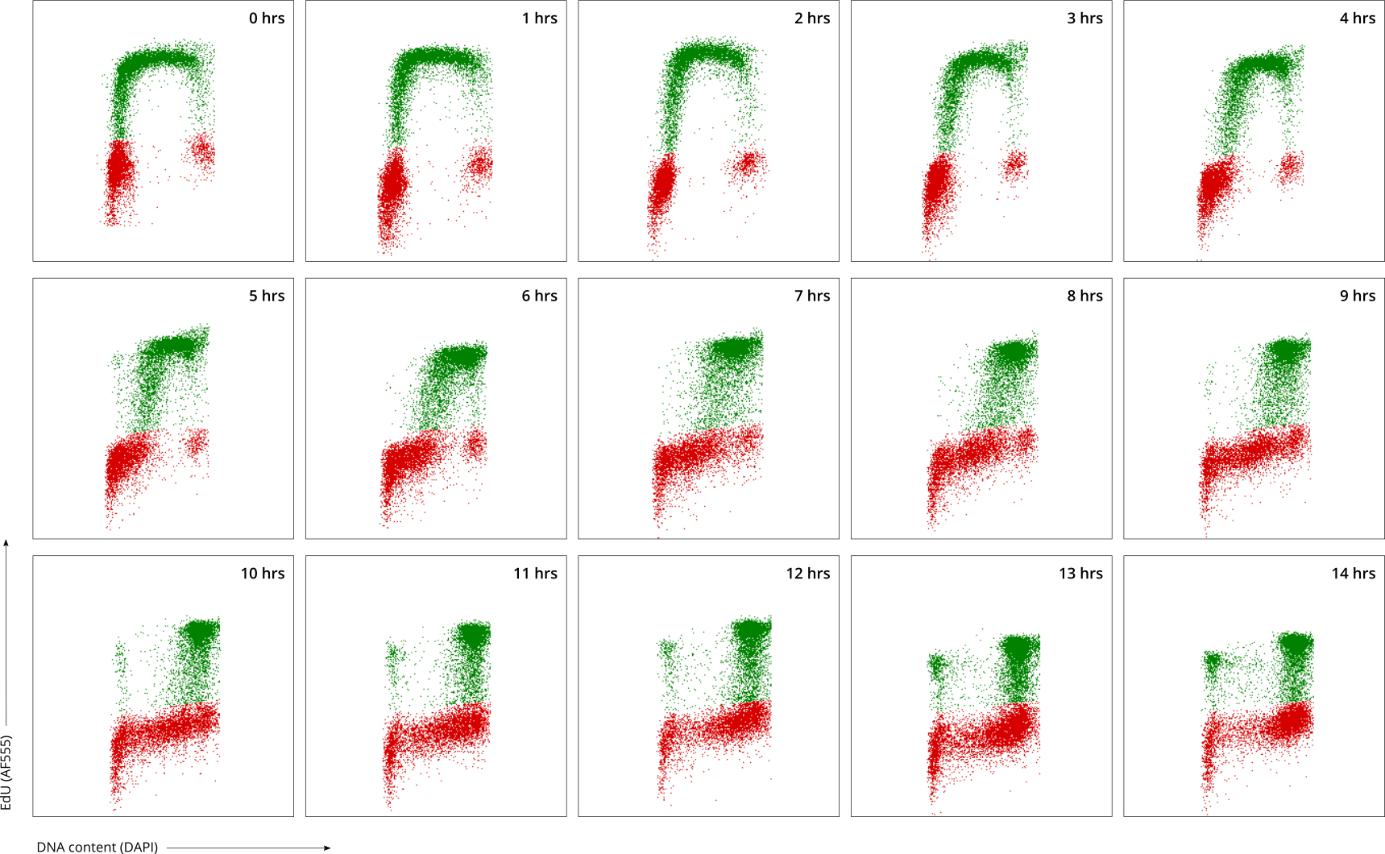
*

**Supplementary Figure S9. EdU pulse-chasing of T lymphocytes over time after exposed to 4 Gy of X-rays.** Bivariate distributions of EdU pulse-labeled T lymphocytes indicated by DNA content (x-axis) and EdU incorporation (y-axis). The green population shows the EdU-positive T lymphocytes. The red population shows the EdU-negative cells. The bivariate profiles of one sample are displayed here. Other samples show similar distributions.

## Supplementary Tables

Supplementary Table 1. Applied method for total cell cycle time (Tc) estimation of non-irradiated CFSE-labeled T lymphocytes. (a) The lymphocyte proliferating index (LPI) was determined for T lymphocytes stimulated with PHA for 24, 48, 72, and 96 hrs. The average LPI of 6 experiments is shown with corresponding standard deviations. (b) The increase in LPI over 24 hrs was calculated for each time point. Subsequently, the average LPI increase over 24 hrs was determined for each experiment, showing the estimated number of divisions made by proliferating T lymphocytes over a time period of 24 hrs. From this, the average cell cycle time needed for one division can be estimated for each experiment, and subsequently, for proliferating T lymphocytes in general.


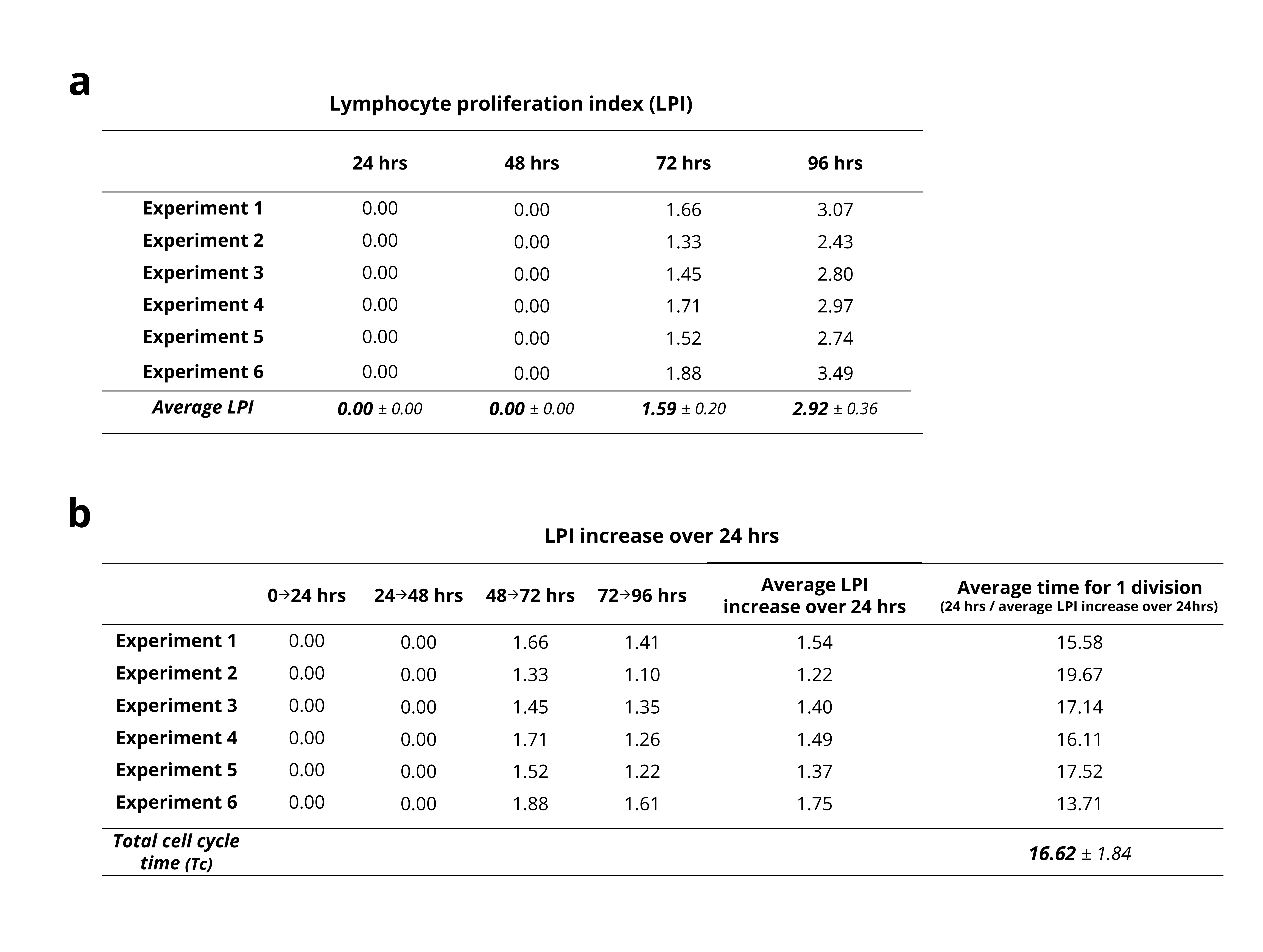


Supplementary Table S2. The average precursor frequency (PF) and lymphocyte proliferation index (LPI) of CFSE-labeled T lymphocytes. The PF and LPI were determined for T lymphocytes cultured in the presence of PHA for 0, 24, 48, 72, and 96 hrs. The CFSE-labeled T cells were exposed to 0, 1, and 2 Gy of 220 kV X-rays before stimulation. The average PF and LPI of 6 independent experiments are shown, with corresponding standard deviations (SD).


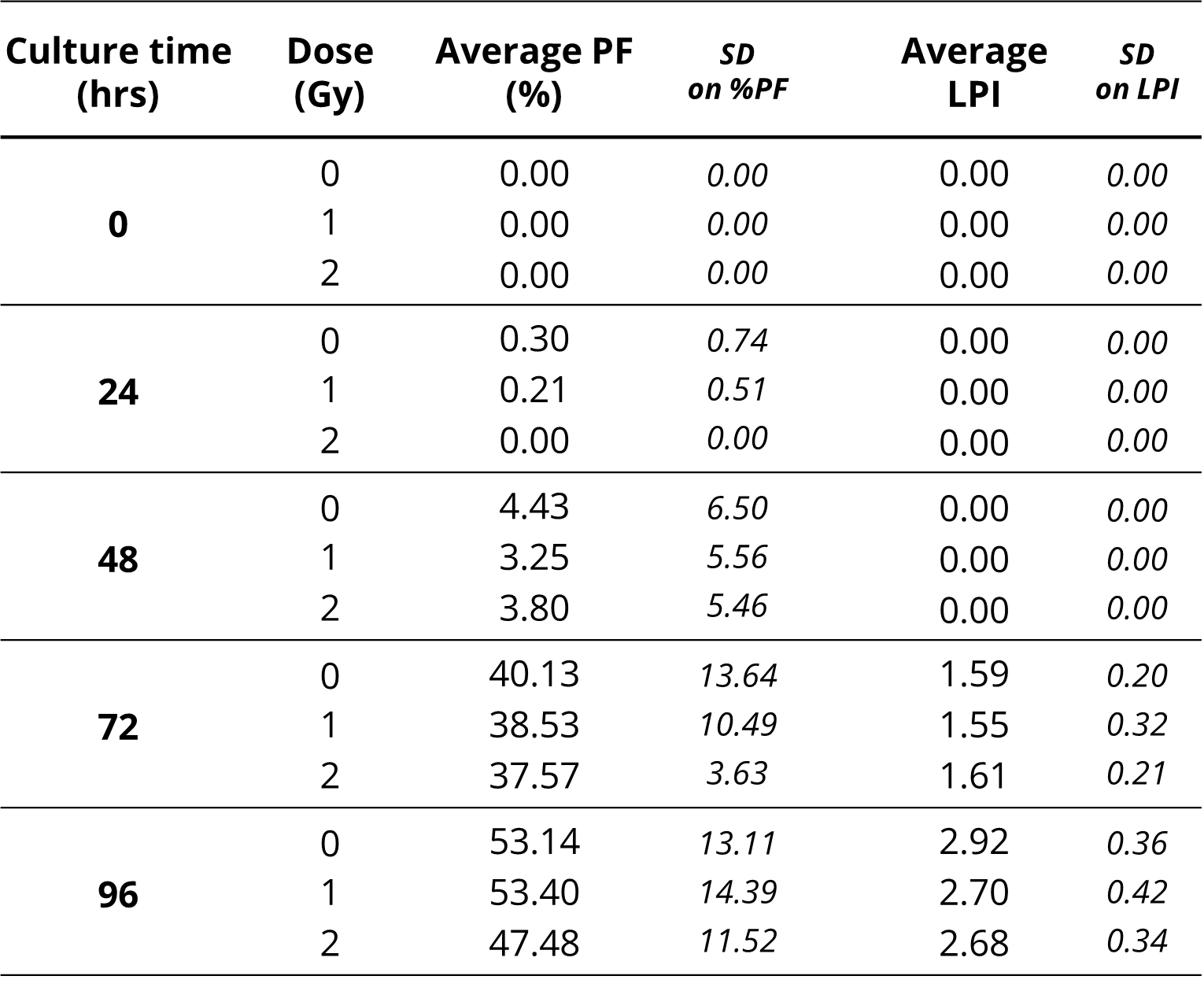

Supplement: Supplementary file 1 — Supplementary Information. [file 41598_2022_11364_MOESM1_ESM.docx]
